# Zebrafish Models of Rare Hereditary Pediatric Diseases

**DOI:** 10.3390/diseases6020043

**Published:** 2018-05-22

**Authors:** Máté Varga, Dorottya Ralbovszki, Eszter Balogh, Renáta Hamar, Magdolna Keszthelyi, Kálmán Tory

**Affiliations:** 1 Department of Genetics, ELTE Eötvös Loránd University, 1117 Budapest, Hungary; r.doresz@gmail.com (D.R.); hamreni@gmail.com (R.H.); 2 MTA-SE Lendület Nephrogenetic Laboratory, 1083 Budapest, Hungary; baloghesztermail@gmail.com (E.B.); keszthelyi0527@gmail.com (M.K.); 3 Ist Department of Pediatrics, Semmelweis University, 1083 Budapest, Hungary

**Keywords:** zebrafish, rare disease models, drug screening

## Abstract

Recent advances in sequencing technologies have made it significantly easier to find the genetic roots of rare hereditary pediatric diseases. These novel methods are not panaceas, however, and they often give ambiguous results, highlighting multiple possible causative mutations in affected patients. Furthermore, even when the mapping results are unambiguous, the affected gene might be of unknown function. In these cases, understanding how a particular genotype can result in a phenotype also needs carefully designed experimental work. Model organism genetics can offer a straightforward experimental setup for hypothesis testing. Containing orthologs for over 80% of the genes involved in human diseases, zebrafish (*Danio rerio*) has emerged as one of the top disease models over the past decade. A plethora of genetic tools makes it easy to create mutations in almost any gene of the zebrafish genome and these mutant strains can be used in high-throughput preclinical screens for active molecules. As this small vertebrate species offers several other advantages as well, its popularity in biomedical research is bound to increase, with “aquarium to bedside” drug development pipelines taking a more prevalent role in the near future.

## 1. Introduction

Rare diseases, namely conditions with incidence rates lower than 1:2000 affect an estimated 350 million people worldwide. More than 7000 such diseases have already been described, and 80% of these are thought to have genetic origins [1,2,3,4]. Approximately one out of 15 infants born worldwide will be affected by a rare hereditary disease during their lifetime [5]. 50–75% of these diseases affect children, and one third of children born with such a condition die before their fifth birthday [1].

The advent of novel methodologies (e.g., next-generation sequencing (NGS)) has made efforts to identify the genetic causes of rare diseases easier, faster and cheaper, yet an accurate molecular diagnosis is still far from trivial with our current knowledge [4]. Disease mapping with current technologies will often yield multiple hits. Many of the flagged alleles are rare variants with unknown effects of genes with known function, or deleterious-looking variants of unknown genes. Deciding which one of these hits is the causative mutation behind the observed phenotype can be a formidable challenge, but often these hard questions can be successfully tackled with the help of model organism genetics [6]. 

Over the past four decades zebrafish has become one of the most in-demand genetic organisms [7,8,9,10]. Researchers have realized early on that this small freshwater fish species bears several characteristics of an ideal vertebrate genetic model organism (it is cheap to maintain, has a small size, is transparent in the embryonic stage, has an external fertilization and a relatively short generation time). But it was the advent of an easy-to-use and ever expanding genetic toolkit that made the zebrafish hugely popular.

The first successful large-scale forward genetic screens [11,12] resulted in a treasure trove of important mutants and demonstrated that this approach can be applied to find mutations with biomedical relevance [13]. Publication of the first detailed genetic maps [14,15,16,17] and sequencing the full diploid zebrafish genome [18] has made the previously challenging and tedious positional mapping of the mutations much more straightforward [19]. Recent systematic efforts, such as the Zebrafish Mutation Project (ZMP) coordinated by the Sanger Institute (UK), aim to mutate every gene in the zebrafish genome (up to date 37,624 alleles of 14,934 genes have been created) [20] and advances in NGS technology offer a far more rapid and straightforward solution for the mapping of novel mutations [21,22]. 

While zebrafish has been used primarily in developmental studies, over the past two decades it has also become one of the most relevant model organisms used in human pathogenetic studies [5,6,23,24,25]. 

**Figure 1:**
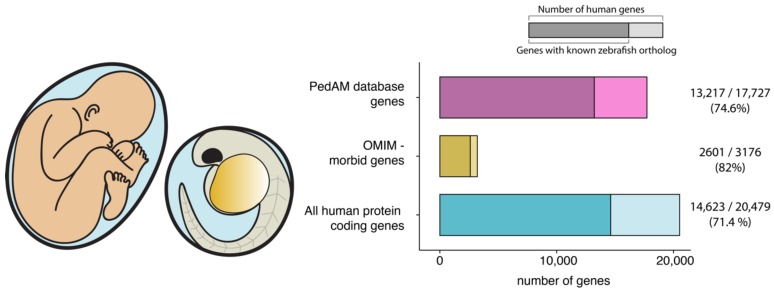
A high level of genetic conservation makes zebrafish an ideal genetic model organism to study pediatric disease. (OMIM–Online Mendelian Inheritance in Man database). Data sources: [18,26]. (Note that the PedAM database contains 4542 unique disease concepts, but the majority of them are associated with multiple genes.).

As a vertebrate organism, zebrafish shares many anatomical features with humans. Furthermore, a high level of genetic conservation can be observed between the two species. Sequencing of the zebrafish genome revealed that 71% of all human proteins and 82% of disease causing ones have a zebrafish ortholog [5,18]. Compared with the genes included in the recently published PedAM database of pediatric disease annotation [26] we found that ~75% (13,217/17,727) of PedAM genes have a clear zebrafish ortholog (Figure 1).

All these advantages, supplemented with an advanced genetic toolkit (see below) make zebrafish uniquely suited for studying human diseases, and for the screening of potential drugs [5,6]. Accordingly, the use of zebrafish features prominently in several large international collaborations (e.g., Undiagnosed Diseases Network (UDN) and Rare Diseases Models and Mechanisms (RDMM)) that aim to study potential disease causing genes with the help of model organisms [6].

## 2. The Zebrafish Genetic Toolkit

Forward genetic approaches, which are used for the identification of mutated genes underpinning specific phenotypes of interest are complemented by reverse genetic approaches, of which antisense morpholino oligonucleotides, morpholinos (MOs) have been the most popular [27]. While the expansion of the zebrafish genetic toolbox has increased the model’s appeal, it is important to keep in mind the limits of the model and some experimental approaches. 

For example, when designing experiments with zebrafish orthologs of particular human disease genes it is worth remembering that due to a whole genome duplication in the Teleost lineage, some human genes have two zebrafish paralogs ([18]). Often, but not always, these paralogs show signs of subfunctionalization ([28,29,30]). Therefore, while for some disease models the knock-down of both paralogs will be necessary, in other cases knocking down a single paralog could give a phenotype that is equivalent to the one observed in human patients. 

### 2.1. Transient Genetic Approaches

Synthetic MO oligos are very stable and can be easily injected into embryos at 1–2 cell stage, where they interfere with gene expression. MOs can bind and mask the translational start site of mRNAs, or can interfere with splicing, in effect creating loss-of-function phenotypes. This straightforward and simple approach became extremely popular among researchers looking for quick assays to test candidate genes from NGS/Genome Wide Association Studies (GWAS), and in numerous studies MO-based experiments and the resulting morphant phenotypes provided the necessary proof to validate the identification of disease genes (Table 1). They can be also used as genetic proof by phenocopy when mapping mutants from forward genetic screens.

Although MO technology was enthusiastically adopted by the zebrafish field and morphant phenotypes were often used in human genetic studies to provide independent proof for the involvement of particular genes in the observed pathologies, the approach has important limitations. For example, MO injections (just like mRNA injections) will have transient effects (see later). But just as important is the fact that MOs can elicit strong, specific p53-dependent effects [31] and recent analysis has also shown that the activation of an innate immune response and off-target miss-splicing are common side effects of MO usage [32]. Furthermore, some early studies of genome-edited lines have raised serious questions about the veracity of many results that were based on MO-effects only ([33]). All these findings resulted in a reconsideration of the use of MOs in the zebrafish field and paved the way to stringent new guidelines [34,35]. 

It would be easy to conclude that a morphant phenotype should be considered specific only if it is able to phenocopy a mutation. However, recent research suggests that the unaltered phenotype in many zebrafish mutants could be the result of either genomic compensation triggered by non-sense mediated decay [36,37] or altered mRNA processing [38,39]. These compensatory effects are not apparent in transcriptional knock-downs, such as MOs and CRISPR-interference (CRISPRi). Overall, the current consensus in the field is to consider MO results specific not only when there is a mutant allele with an identical phenotype, but also if the injection of the MO into the mutant background has no visible phenotypic effects, even though there are differences between the phenotypes of the morphants and mutants of a particular gene. 

If a *bona fide* mutant for the gene of interest is not available, it is of utmost importance to use proper controls when working with MOs. These include the use of multiple MOs, their careful titration and the demonstration that the gene of interest is successfully targeted (either by Western blot if antibodies are available, or at least by RT-PCR to monitor altered splicing in the case of a splice blocking MO). 

Similarly to MOs, in vitro synthesized mRNAs can be introduced easily into early stage embryos. These methodologically easy and quick gain-of-function experiments have been widely used to decipher the role of genes during early development. As mRNA injection can be used to express dominant-negative or constitutively active constructs, too, in combination with MOs, it has been successfully applied for epistasis analysis experiments [40]. (Co)injection of mRNAs has been also widely used as genetic proof to validate the specificity of morphant and mutant phenotypes.

### 2.2. Stable Genetic Approaches 

Only with the application of TALEN-based [114,115,116] and, more recently, CRISPR/Cas9-based genome editing technologies in zebrafish [117,118,119,120,121,122] has the use of MOs seen a decline. These new methodological approaches have already revolutionized zebrafish genetics [123] and provided independent means to test the veracity of the morphant phenotypes (see above). While most of the novel genome edited lines are loss-of-function alleles that arise due to the indel mutations resulting from erroneous non-homologous end joining (NHEJ) DNA repair mechanisms, efforts have been made to create precise knock-in alleles exploiting the alternative, homologous recombination (HR) repair pathway [124,125,126]. While the excitement caused by these early results seemed justified, later results suggested that the knock-in efficiency is highly locus- (and template-) dependent.

Of the existing programmable nuclease techniques TALENs, albeit slower and more expensive to assembly, are usually considered superior, due to their higher specificity. The off-target effects of CRISPR can be, however, considerably reduced with the right choice of sgRNAs and with the use of rationally engineered Cas-variants [127,128].

As the problems with MOs became apparent, many people opted to complement or supplement MO studies with the description of “crispant” phenotypes (see Table 1). In crispants CRISPR/Cas9 technology was used to introduce mutation(s) with gene-specific sgRNA. In case of embryonic- or larval-lethal mutations, this approach could provide a quick and cost-effective way to test the function of the genes of interest. Due to the very nature of this method, however, most embryos will be highly mosaic for the mutations they carry, and only careful analysis can reveal if they indeed have biallelic mutations in most of their cells. Therefore, we should tread carefully and only accept crispant phenotypes as specific if constitutive mutants show the same phenotype. (Ideally, one should aim to conduct studies in F2 or F3 generations, where the possible confounding effects of off-target mutations can be minimized). It will be also important to compile databases of proven and effective sgRNA target sequences with low off-target effects, so that targeting of particular genes with CRISPR-based methods can become more standardized [129].

The advantages of *bona fide* mutants over morphants and crispants are numerous, however, as mentioned above, the phenomenon of genetic compensation can hinder the characterization of mutant phenotypes [36,37,38,39]. 

The transparency of zebrafish embryos and larvae has been long considered one of the most advantageous attributes of the model. Organs, tissues or specific cells can be labeled with fluorescent dyes and markers and followed in vivo under a microscope. Transgenic lines have been instrumental in characterizing the effects of specific mutations (e.g., [44,52,58,130]), highlighting the power of this approach. The modular, easy-to-use “Tol2-kit”, based on the Gateway technology has made the creation of transgenic lines a mundane task [131]. Efficient transgenesis techniques have been also used for enhancer-trap and gene-trap screens [132,133], and a wide array of tissue-specific Gal4 and CreERT2 lines have been established, paving the way for intricate genetic manipulations [134,135,136]. 

Finally, the combination of transgenesis and genome editing techniques enabled researchers to create the conditional knock-out methodology that previously eluded the zebrafish field [137,138].

## 3. Modeling Disease with Homologs and Phenologs

In the past few decades zebrafish has emerged as a powerful model of congenital disorders (Table 1). This development is partly due to the advantages of the model (embryonic development is fast and external, therefore, the emergence of particular impairments can be followed effectively in real time and phenotypes can be identified early), but also to the limitation of the tools used. For example, although MOs can bring almost complete knock-down, their effect is temporary, and after 3–5 days it diminishes sharply [139]. mRNAs are equally unstable (or even more so), thus their effect is limited to the first 2–3 days of development. Importantly, unlike many disease alleles in humans, the majority of zebrafish alleles for the respective genes isolated in previous screens or created recently with novel editing methods are embryonic-lethal null-alleles. 

Despite these limitations MO-based knockdowns (lately backed up by crispant phenotypes, resulting from the injection of CRISPR/Cas9 RNPs into embryos) have been very successfully used in identifying driver genes for particular diseases. In a recent study of DiGeorge syndrome fish models have been essential to demonstrate that haplo-insufficiency of *CRKL* is the main cause of the kidney pathologies observed in patients with this syndrome [44].

Due to the high levels of genetic and anatomical conservation between the two species, zebrafish models often display highly similar phenotypes to the human condition. For example, models of coloboma, generalized arterial calcification of infancy (GACI), X-linked adrenoleukodystrophy (ALD), Duchenne’s muscular dystrophy or Dravet syndrome all display features that are highly similar to the characteristics of the human pathologies (see Table 1 for references).

In certain cases prior in-depth knowledge about zebrafish development helps to create highly informative disease models through orthologous phenotypes, or phenologs [140]. For example, zebrafish models have been instrumental in deciphering the role of several genes in ciliopathies, such as Bardet-Biedl Syndrome (BBS) or Joubert syndrome (Table 1). The popularity of zebrafish in ciliopathy research can be at least partly explained by the fact that dysfunction of the cilia results in easily recognizable developmental phenotypes, including curved body axis, hydrocephalus and laterality defects [141].

Another excellent example for the use of phenologs in modeling pediatric disorders is fibrodysplasia ossificans progressiva (FOP). In-depth knowledge about the molecular mechanism of early dorso-ventral (DV) patterning in zebrafish development, including the phenotype of ventralized embryos, has been helpful both in the identification of the causative mutations of FOP [63,64] and that of putative drugs [142]. 

Constitutively active mutations, such as the ones observed in *ACVR1* in patients with FOP, can be modeled using mRNA injections and transgenic approaches. This approach can substitute or complement transient expression studies when necessary [65]. Overexpression experiments can be also informative in modeling microduplications or trisomies [70,90]. 

Finally, although MOs are usually injected in significant excess to obtain functional knock-downs, they can be also titrated to suboptimal concentrations to mimic the additive effects of hypomorphic mutations. A nice example for this approach is provided by the modeling of the Charcot-Marie-Tooth syndrome, where the “mutational burden” hypothesis of neuropathy genes was tested [53].

## 4. Drug Discovery Using Zebrafish

Its small size, allowing for semi-high-throughput screening, has made zebrafish a prominent model in drug screens over the past decade. In addition, due to the aforementioned high level of genetic conservation between zebrafish and humans, several drugs have similar targets (and thus similar effects) in both species. It is, therefore, no wonder that over the past decade zebrafish has emerged as the model organism of choice for high-throughput screening of chemical libraries for potential drugs [5] and several compounds picked up in these screens have made their ways into clinical trials [143]. 

Several models of pediatric disorders have been used in such screens, and these experiments confirm that both homologous and orthologous phenotypes can be successfully used in drug discovery/testing experiments (Table 2). For example, a recent model of childhood-onset parkinsonism-dystonia, characterized by mutations in a Mn-transporter, was successfully used to show that the symptoms of the disease can be ameliorated using Na_2_CaEDTA as a chelator–and this treatment also alleviates the patients’ symptoms [54]. 

Identification of the causative FOP mutations in the *ACVR1* gene and the validation of the orthologous ventralized phenotypes in zebrafish led to the later discovery of dorsomorphin and its derivatives [142]. These molecules with dorsalizing effects are currently being tested in clinical trials for FOP treatment. An even more impressive recent “aquarium to bedside” story involves a zebrafish model of Dravet syndrome: a high-throughput drug screen identified clemizole and lorcaserine as potential drugs with anti-serotonin effects. These drugs have been approved by the FDA earlier as an antihistamine and a weight-loss aid, respectively. It was, therefore, possible to register them as potential treatment without having to repeat the expensive, time-consuming clinical trials that enabled them to be approved. When applied directly to patients with Dravet syndrome, these repurposed drugs outperformed conventional anticonvulsants [144].

While zebrafish studies will not always substitute pre-clinical tests in mammalian models, they can save time and money by pre-filtering the compounds that enter the more advanced phases of drug development ([5,145]).

## 5. Outlook

Overall, despite the aforementioned limitations that can be overcome with proper controls or novel technologies, zebrafish models of rare pediatric diseases (and diseases in general) are set to probably become even more important assets of preclinical research and drug discovery in the coming years. We can almost certainly expect a proliferation of the repurposing studies of FDA-approved drugs, as zebrafish is the ideal model to conduct such studies. The increasing relevance of fish models will be also apparent in the study of childhood leukemias and other forms of cancer (for comprehensive reviews on this subject see [148,149,150]) and zebrafish “avatars” for the development of personalized chemotherapies could also become prominent in the near future ([151,152,153]).

With the proliferation of base-editor tools that are often based on synthetic versions of Cas-nucleases with altered PAM recognition sites, genome editing has entered a new phase, often referred as “CRISPR 2.0” ([154,155,156,157]). We can routinely engineer A to G and C to T transitions with high precision in the genome, and as zebrafish has been at the forefront of the CRISPR-revolution, it is almost certain that the coming years will see a proliferation in the use of these base editors. This technological breakthrough will help us create exact or almost exact mimics of hypomorphic human disease alleles (instead of nulls), making the new disease model strains even more relevant in examining particular aspects of human pathologies. We can also expect the proliferation of “humanized” zebrafish lines as well, where zebrafish carrying mutations in particular genes are supplemented with a transgenic cassette expressing the human ortholog of the gene ([158]). Zebrafish models created with precision base-editing methods could also help understanding how rare variants of Mendelian genes contribute significantly to complex disease phenotypes, as suggested by recent research [159].

Finally—and somewhat counterintuitively—even the fact that zebrafish mutants often lack an overt phenotype could be exploited to better understand human diseases [39]. Recent studies suggest that in certain unaffected individuals who are homozygotes or compound heterozygotes for null alleles, the effect of these loss-of-function alleles can be negligible [160]. The study of mutant zebrafish strains that are phenotypically normal could reveal how their robustness is achieved by translational plasticity [161], providing important insights into the context dependency of genetic risk factors.

## Figures and Tables

**Table 1:** A representative list of existing zebrafish pediatric disease models.

Disease Name	ICD-10	Genes Targeted in Models	OMIM IDs	Model Type	References
Diseases of the blood and blood forming organs					
Blackfan-Diamond anemia	D61.0	*RPS19*, *RPL11*, *RPS7*	105650, 612562, 603658	MO, mutant	[41,42,43]
DiGeorge syndrome	D82.1	*SNAP29*, *AIFM3*, *CRKL*	604202, 617298, 602007	MO, crispant	[44]
Reticular dysgenesis	D81.0	*AK2*	267500	MO	[45]
Sideroblastic anemia (AR)	D64.0	*SLC25A38*	205950	MO	[46]
X-linked sideroblastic anemia	D64.0	*ALAS2*	300751	mutant	[47]
Endocrine and metabolic diseases					
Batten disease (Juvenile neuronal ceroid lipofuscinosis)	E75.4	*CLN3*, *TPP1*	204200, 204500	mutant, MO	[48,49]
Menkes disease	E83.0	*ATP7A*	309400	mutant	[50]
Nephropatic infantile cystinosis	E72.0	*CTNS*	219800	mutant	[51]
X-linked adrenoleukodystrophy (ALD)	E71.3	*ABCD1*	300100	mutant	[52]
Diseases of the nervous system					
Charcot-Marie-Tooth syndrome	G60.0	*MFN2*, *GDAP1*, *ABHD12*, *MED25*, *HSPB1*, *WNK1*	608507, 606598, 613599, 610197, 602195, 605232	MO	[53]
Childhood-onset parkinsonism-dystonia		*SLC39A14*	617013	mutant	[54]
Dravet syndrome	G40.4	*SCN1A*	182389	mutant	[55]
Duchenne muscular dystrophy	G71.0	*DMD*	310200	mutant	[56]
Generalized epilepsy with febrile seizures-plus	G40.3	*STX1B*	616172	MO	[57]
Spinal muscular atrophy	G12	*SMN1*	600354	MO, mutant	[58,59]
Diseases of the circulatory system					
Dilated cardiomyopathy	I42.0	*BAG3*	603883	MO, transgenic	[60,61]
Timothy syndrome	I45.8	*CACNA1C*	601005	MO	[62]
Diseases of the musculoskeletal system					
Fibrodysplasia ossificans progressiva (FOP)	M61.1	*ACVR1*	135100	mRNA, transgenic	[63,64,65]
Vasculitis due to ADA2 deficiency	M30.8	*ADA2*	615688	MO	[66]
Diseases of the genitourinary system					
Polycystic kidney disease (PKD)		*PKD1*, *PKD2*	173900, 613095	MO, mutant	[67,68,69]
Congenital malformations					
16p11.2 microdeletion/microduplication syndrome		*KCTD13*	608947	MO, mRNA	[70]
3MC syndrome	Q87.8	*COLEC11*, *MASP1*	265050, 257920	MO	[71]
Autosomal recessive polycistic kidney disease	Q61.1	*DZIP1L*	617610	MO, mutant	[72]
Axenfeld-Rieger syndrome	Q13.8	*PITX2*	180500	mutant	[73]
Bardet-Biedl syndrome (BBS)	Q87.8	*BBS1*, *BBS2*, *BBS4*, *BBS5*, *BBS6*, *BBS7*, *BBS8*, *BBS10*, *BBS11*, *BBS12*, *CCDC28B*	20991, 600374, 605231, 615981, 615983, 615984, 615985, 615987, 615988, 615989, 610162	MO,	[74,75,76,77,78,79]
Cardiofaciocutaneous syndrome	Q87.8	*MEK1*	615279	mRNA	[80]
Coloboma		*GDF6*, *MAB21L2*, *PTCH1*, *YAP1*	601147, 615877, 601309, 120433	mutant	[81,82,83,84]
Congenital anomalies of kidney and urinary tract (CAKUT)		*DSTYK*	612666	MO	[85]
CHARGE syndrome	Q87.8	*CHD7*	608892	MO, mutant	[86,87]
COACH syndrome	Q04.3	*MKS3*/*TMEM67*	216360	MO	[88,89]
Down syndrome	Q90	*21q22.3*	190685	mRNA	[90]
Dyskeratosis congenita	Q82.8	*DKC1*, *NOLA3*/*NOP10*	305000, 224230	MO, mutant	[91,92]
Galloway-Mowat syndrome	Q04.3	*OSGEP*, *TPRKB*	617729, 617731	crispant	[93]
Generalized arterial calcification in infancy (GACI)	Q28.8	*ABCC6*, *ENPP1*	614473, 208000	MO, mutants	[94,95,96]
Infantile nephronophthisis	Q61.5	*ANKS6*	615382	MO	[97]
Joubert syndrome	Q04.3	*JBTS1*/*INPP5E*, *JBTS2*/*TMEM216*, *JBTS3*/*AHI1*, *JBTS5*/*CEP290*, *JBTS7*/*RPGRIP1L*, *JBTS8*/*ARL13B*, *JBTS9*/*CC2D2A*, *JBTS10*/*OFD1*, *ARMC9*	213300, 608091, 608629, 610188, 611560, 610688, 612291, 612285, 300804, 617612	MO, mutant	[89,98,99,100,101,102,103,104,105]
MARCH syndrome		*CEP55*	610000	MO, crispant	[106]
Pontocerebellar hypoplasia (1B)	Q04.3	*EXOSC3*	614678	MO	[107]
Primary ciliary dyskenesia	Q34.8	*ARMC4*, *CCDC40*, *ZMYND10*	615451, 613799, 615444	MO, mutant	[108,109,110]
Robinow syndrome (AD)	Q87.1	*WNT5A*	180700	mRNA	[111]
Senior-Løken syndrome	Q61.5	*SDCCAG8*	613615	MO	[112]
Spondyloepimetaphyseal dysplasia	Q77.7	*NANS*	610442	MO	[113]

**Table 2:** Some examples for the use/test of drugs with human relevance in zebrafish disease models.

Syndrome	Drug/Small Molecule Used	Target/Function	References
Aortic coarctation	GS4012	VEGF inducer	[130]
Blackfan-Diamond anemia	PF477736	CHK1 inhibitor	[146]
Childhood-onset parkinsonism-dystonia	Na_2_CaEDTA	Mn chelator	[54]
Dravet syndrome	clemizole	Serotonin modulators	[55,144]
	lorcaserin		
Duchenne muscular dystrophy	Ataluren (PTC124)	Translational readthrough agonist	[147]
Fibrodysplasia ossificans progressiva (FOP)	Dorsomorphin (and derivatives)	BMP Type 1 Receptor inhibitor	[142]
Generalized arterial calcification in infancy (GACI)	Etidronate	PPi analog	[95]
Sideroblastic anemia (AR)	Glycine and folate	supplement	[46]
Spondyloepimetaphyseal dysplasia	Sialic acid	supplement	[113]
